# Visual masking: past accomplishments, present status, future developments

**DOI:** 10.2478/v10053-008-0010-7

**Published:** 2008-07-15

**Authors:** Bruno G. Breitmeyer

**Affiliations:** Department of Psychology, University of Houston

**Keywords:** masking, neural networks, nonconscious/conscious processing, object perception

## Abstract

Visual masking, throughout its history, has been used as an investigative tool in exploring the temporal dynamics of visual perception, beginning with retinal processes and ending in cortical processes concerned with the conscious registration of stimuli. However, visual masking also has been a phenomenon deemed worthy of study in its own right. Most of the recent uses of visual masking have focused on the study of central processes, particularly those involved in feature, object and scene representations, in attentional control mechanisms, and in phenomenal awareness. In recent years our understanding of the phenomenon and cortical mechanisms of visual masking also has benefited from several brain imaging techniques and from a number of sophisticated and neurophysiologically plausible neural network models. Key issues and problems are discussed with the aim of guiding future empirical and theoretical research.

## Brief Coda to a Long History

Masking always has been a way of investigating the temporal properties of processes underlying visual sensations and perceptions. It has been particularly important in the studying the microgenesis of object perception. I cannot review all of the related accomplishments of the past. For that I refer the reader to Chapter 1 of the 2^nd^ edition of our book, *Visual Masking* (Breitmeyer & Öğmen, 2006). It amply reviews the history of masking from the late 19^th^ century to the middle of the 20^th^. Looking at the wider span of about 140 years up to the present, one can, however, discern some interesting features, transitions, or phases in the study of masking. Toward the turn of the 19^th^ century, masking was viewed as a way of exploring interactions thought to occur anywhere along the visual tract, from lateral interactions in the retina to cortical processes underlying object cognition and consciousness. With the ascendance of behaviorism some decades later, the topic of cognition and especially consciousness took a nosedive toward oblivion. With the exception of Piéron’s (1935) and Werner’s (1935) more impressionistic and phenomenological accounts, visual masking studies concentrated on parametric variation of stimulus properties, threshold measurements and quantification of the functional properties of masking. Particularly good examples of this kind of work were the classical studies on masking of light performed by Crawford (1947) and on metacontrast by Alpern (1953) toward the middle of the 20^th^ century. Both investigations and their immediate offshoots focused on pro-cesses – early light and dark adaptation, interactions among rod and cone activations – that were deemed to occur at early, peripheral levels. Neither was remotely concerned with higher brain processes related to cognition or consciousness. While masking by light is largely confined to peripheral, most likely retinal, processes (Battersby, Oesterreich, & Sturr, 1964), we now know that the crucial aspects of metacontrast and pattern masking are determined by cortical interactions. Since the 1960s very few studies were conducted on masking by light, and none that I know of since Cogan’s (1989, 1992) studies in the late 1980s and early 1990s. In contrast, pattern masking and metacontrast studies retained their currency to the present. Why? 

I believe three trends in scientific outlook merged mid century to promote continued interest in, among many other topics, *pattern* masking. Because they specify and actualize a single or a few constellations of features from among a vastly larger set of possibilities, patterns are organized physical or mental entities that convey information. Within that context, one trend was the theory of communication (Shannon & Weaver, 1948), which formalized a rigorous mathematical definition of information in terms of bits. In turn this formalization could be wedded readily with a second concurrent formalization in computational science and artificial intelligence (Turing, 1950). The third was the pioneering work of Hebb (1949) attempting to reconcile phenomenological Gestalt and functional “connectionist” approaches in a plausible neural-network model of the organization of mind and its perceptual and cognitive control of behavior. The imprint of the former influence was clearly left on the pioneering works of Cherry (1953), Broadbent (1958) and Moray (1959) on the role and properties of attention in various “capacity-limited” sensory “channels” of communication, and with respect to masking on the information-processing approaches to visual cognition, with all its “parallel” and “serial” processors, adopted from the early 1960s through 1970s by Averbach and Coriell (1961), Sperling (1963), Scheerer (1973), and Turvey (1973). Additionally, in the late 1950s and early 1960s artificial intelligence spurred, among other things, development of computational models of perception and pattern recognition such as Rosenblatt’s (1958) Perceptron and Selfridge’s (1959; Selfridge & Neisser, 1960) Pandemonium. And Hebb’s (1949) related work on physiologically plausible neural networks of perception anticipated the first attempts around 1970 at providing quantitative neural network models of pattern masking by Weisstein (1968) and by Bridgeman (1971). What I consider to be an important transitional approach to masking was the work of Bachmann (1984, 1994), which appeared at about the same time as the first edition of my book on visual masking highlighting the dual-channel, sustained-transient approach to masking (Breitmeyer & Ganz, 1976). All of the approaches up to that time were of course interested at least implicitly in giving plausible accounts of pattern recognition and other perceptual phenomena. But Bachmann, by incorporating in his neural network model not only the retino-cortical activations providing the *contents* of perceptions but explicitly also the retino-reticular-thalamic activations that play such a crucial role in regulating the state of consciousness, reinstated consciousness and phenomenology in their rightful place alongside purely functionalist descriptions of masking phenomena. I believe that in spirit this approach has been vindicated by the current interest in masking as a way of exploring the neural correlates of conscious and unconscious vision (NCCs and NCUs). 

## What Now?

A lull in theoretical modeling of masking and somewhat also in empirical developments followed Bachmann’s work until roughly the 1990’s, which inaugurated most of what I deem to be “the present” in visual masking research. What have been some of the chief contributions to masking research in this present time period? Of course, some of these were theoretical. However, other equally important ones were methodological and empirical, often closely allied to the theoretical.

### Direct parameter specification and masked priming

 In the late 1980s and early 1990s, a new methodological application of metacontrast masking evolved in the context of the theory of direct parameter specification (DPS). Formulated by the Bielefeld group under the direction of Odmar Neumann, DPS took the findings originally reported by Fehrer and Raab (1962), that a fully masked target could activate processes that facilitated response times in a simple detection task, one step further by arguing and showing that a suppressed target could additionally prime sensori-motor pathways specified by sophisticated figural properties of the subsequent mask stimulus. This is an important result for several reasons. For one it maps neatly onto Milner and Goodale’s (1995) recent theoretical reconceptualization of the dorsal and ventral cortical pathways in terms of the vision for action and the vision for perception systems. Dearer and nearer to my theoretical heart, it also provided a ready and powerful way of investigating the types and levels of unconscious or preconscious visual information processing, a topic that has occupied my research efforts increasingly in the last few years (Breitmeyer, Öğmen, & Chen, 2004; Breitmeyer, Ro, & Singhal, 2004; Breitmeyer, Öğmen, Ramon, & Chen, 2005). More on that later. 

### Four-dot and common-onset masking

During the 1993 meeting of the Psychonomics Society held in Washington, D. C., I had the pleasure of exchanging ideas with Vince Di Lollo on several occasions. On one occasion Vince enthusiastically described the four-dot and common-onset masking techniques (Bischof & Di Lollo, 1995; Di Lollo, Bischof, & Dixon, 1993) and their implications for – in his terms – a fundamentally new conceptualization of masking in terms of downward influences from higher-level processes instead of low-level contour interactions. I was skeptical and privately dismissed his enthusiasm as heady overexcitement. After all, I thought, Naomi Weisstein, Charlie Harris, and their collaborators (Weisstein & Harris,1974; Williams & Weisstein, 1978, 1981) had already demonstrated a higher-level, object-superiority effect in metacontrast; so what’s the deal? Nonetheless, as Vince reminded me at the recent ASSC9 meeting at Caltech, during another of our encounters, perhaps the long walk we took along the Potomac, I suggested he try to relate his ideas to the notion of re-entrant activation; and I referred him to Edelman’s book, *Neural Darwinism*. Re-entrant activation, central to the theoretical thinking of a number of current visual and cognitive neuroscientists (Edelman, 1987; Posner, 1994; Zeki, 1993) is also a central theme in the theory of object-substitution masking (Enns, 2004; Enns & Di Lollo, 1997; Di Lollo, Enns, & Rensink, 2000); and I will argue later that it also will have to be incorporated into other neural network models that make claims to physiological realism. Just as Bachmann’s model of perceptual retouch (PR) – which by the way is a form of object substitution – placed the spotlight on the underadvertised existence of the retino-reticular-thalamic activations, so does object-substitution masking highlight the important roles of heretofore underadvertised yet massive reentrant pathways in the cortical visual system. More on that later also.

### Neuroscientific approaches to masking

 The first neuro- and electrophysiological studies of masking go back nearly four decades. I will not review all of the studies that have been conducted since then; such a review is found in Chapter 3 of our forthcoming book on visual masking (Breitmeyer & Öğmen, 2006). I will highlight the few that, in my opinion, are most revealing in relation to metacontrast and para-contrast masking. Of the older studies, the studies by Schiller and Chorover (1966), Vaughan and Silverstein (1968), and Schwartz and Pritchard (1981) recording human cortical visual evoked potentials (CVEPs) and Bridgeman’s (1980) studies of single cortical cells in monkey all indicate that it is the variations of the later response components of the V1 cortical response which correlate with visibility of a target during metacontrast. When I read these studies, I took their results as confirming the sustained-transient channel approach to masking. According to that model, one would expect suppression of cortical responses to occur in the longer-latency sustained channels, which I assumed were responsible for generating the longer latency or late CVEP components. In gist I believe this is still correct, but not in detail. The reason is that the original dual-channel approach was developed within a feedforward framework. More recent neurophysiological results, however, seriously question this framework. 

**Figure 1.**
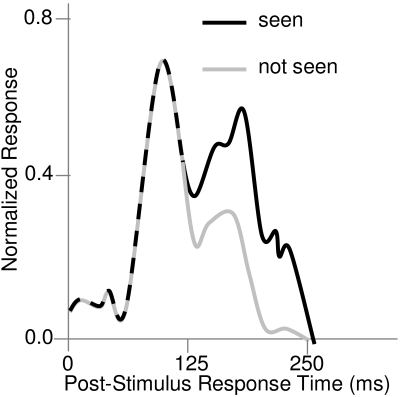
Post-stimulus multi-unit response magnitude functions obtained from V1 monkey neurons when a stimulus is perceived/ seen and when it is not perceived/seen. (Adapted from Lamme, Super, Landman, Roelfsema, & Spekreijse, 2000)

According to Lamme and coworkers (Lamme, 1995; Lamme & Spekreijse, 2000; Lamme, Super, Landman, Roelfsema, & Spekreijse, 2000; Super, Spekreijse, & Lamme, 2001), the late V1 response component, as shown in Figure 1, is associated with percept-dependent activity and is due to re-entrant activation from higher cortical regions, while the early component, associated with stimulus-dependent activity, is due to the afferent, feedforward sweep of activation. Thus in detail these late components are not due to long-latency afferent or feedforward drive, as I had thought, but rather due to re-entrant activation from higher cortical visual areas. While I still believe the gist that metacontrast suppression is exerted on the sustained parvocellular-dominated cortical pathway (see below), I also believe that it occurs at the feedback/reentrant level rather than the feedforward level.

**Figure 2.**
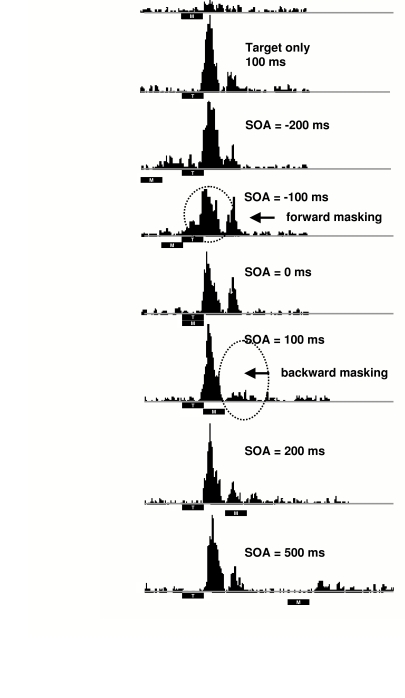
Multi-unit recordings from upper layers of area V1 of rhesus monkey. Note as indicated by dashed ovals a) optimal suppression of the early onset response component at a paracontrast SOA of -100 ms and b) optimal suppression of the later response component at a metcontrast SOA of 100 ms. (From Macknik & Livingstone, 1998)

I believe this view is also consistent with the some of the recent results reported by Macknik and Livingstone (1998). They showed (see Figure 2) that metacontrast suppresses a later target-response component which they associated with the offset of the target, whereas it had virtually no effect on the early response component associated with target onset. In contrast, when a paracontrast mask was applied, powerful suppression of the early response component occurred along with some suppression of the later component. What is one to make of these findings? While other interpretations are clearly possible, my preferred one runs as follows: First, paracontrast exerts its effects primarily on the early feedforward activity and secondarily on the late reentrant activity, since this late activity “feeds on” the feedforward drive. That is to say, since the feedforward drive in V1 is suppressed by paracontrast, the later cortical levels in the feedforward sweep are also activated less; hence the reentrant feedback emanating from them will be weaker, leading also to a suppressed late V1 response component. Second, metacontrast exerts its suppressive effects only on the late, reentrant activity.

**Figure 3.**
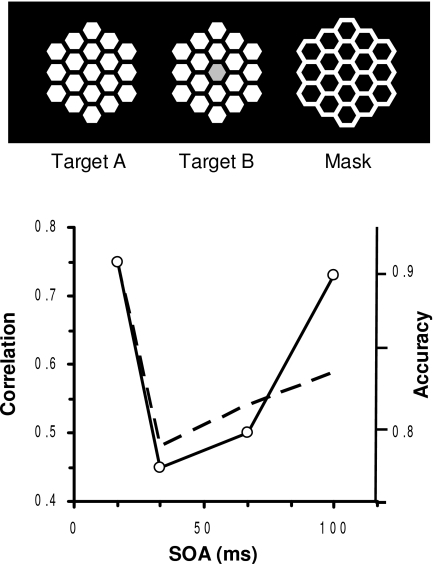
Upper panel: “Honeycomb” target and mask stimuli. Lower panel: Correlation, derived from the fMRI results of the same observer, between activity in V1 level and the fusiform- gyrus (FG) level of cortical processing as a function of the SOA between the targets and the mask. (From Haynes, Driver & Rees, 2005)

Based on their results and on the above reasoning, Macknik and Livingstone (1998) developed what I believe to be currently the most effective masking method, namely, the standing-wave illusion, for rendering stimuli invisible. In this method a mask appears about 100 ms before the target, which in turn is followed about 50 ms by the mask, followed 100 ms by the target and so on. Basically the target and mask are presented at optimal para- and metacontrast SOAs throughout the presentation (see Figure 5 below), thus giving the target a “double masking whammy” by suppressing first its feedforward activity and then in addition the (already weakened) re-entrant activity. While this method produces very powerful suppression of target visibility that correlates well with brain imaging (fMRI) findings (Tse, Martinez-Conde, Schlegel, & Macknik, 2005), it renders difficult any interpretations of results in terms of either para- or metacontrast effect alone. However, thanks to the work of Haynes, Driver, and Rees (2005) we do have brain imaging results that were obtained with an isolated metacontrast effect. What their findings show (see Figure 3) is that the functional correlation between earlier (V1) and later (fusiform gyrus) areas in visual cortex is suppressed by the metacontrast mask. In view of what I have outlined so far above, I suspect that the disruption of connectivity is due to a reduction of reentrant feedback from higher to lower areas. Is there independent, convergent evidence for this feedforward and reentrant scheme of para- and metacontrast?

### TMS and visual masking

**Figure 4.**
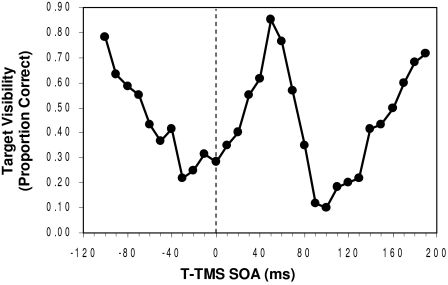
Visibility (in proportion correct identification) of the target as a function of the onset asynchrony separating it from the TMS pulse. Negative SOAs: TMS precedes target; positive SOAs: TMS follows target. (Adapted from Corthout, Uttl, Ziemann et al., 1999).

A series of experiments conducted by Corthout et al. (Corthout, Uttl, Walsh, Hallett, & Cowey, 1999; Corthout, Uttl, Ziemann, Cowey, & Hallett, 1999) demonstrated masking effects of transcranial magnetic stimulation (TMS) on foveal targets consisting of individual letters. Figure 4 shows typical results (Corthout, Uttl, Ziemann et al., 1999) of TMS masking as a function of the SOA between the TMS pulse and the visual target. Negative and positive SOAs indicate that the TMS onset respectively preceded and followed the onset of the visual target. Masking magnitude is indicated by the proportion of correct identifications of the target letters, with lower proportions corresponding to stronger masking. Note that two masking maxima were obtained, one at an SOA of –30 ms and the other at an SOA of 100 ms. Corthout, Uttl, Ziemann et al. (1999) concluded – rightly in my opinion – that these two maxima corresponded to the TMS-induced disruption of two processing intervals, the former corresponding to the early feedforward activation of cortical neurons and the latter to activation depending on re-entrant feedback from higher cortical visual areas. This interpretation dovetails nicely with the aforementioned proposal of Lamme and co-workers (Lamme, 2001; Lamme & Spekreijse, 2000; Lamme et al., 2000; Super et al., 2001) regarding an early feedforward and stimulus-dependent component and a later re-entrant and percept-dependent component of V1 neural responses.

The two TMS masking maxima found by Corthout et al. (Corthout, Uttl, Walsh et al., 1999, Corthout, Uttl, Ziemann et al., 1999) are very reminiscent of paracontrast and metacontrast maxima obtained with visual masks. In fact, below I argue that the two TMS and the two visual mask maxima indicate suppression of the same response components. This view is consistent, on the one hand, with Macknik and Livingstone’s (1998) aforementioned finding that paracontrast suppresses the early response component of V1 neurons and, on the other, with the finding also mentioned above that backward pattern masking suppresses the later response components (Andreassi, De Simone, & Mellers, 1975; Bridgeman, 1980; Lamme et al., 2002; Schiller & Chorover, 1966; Schwartz & Pritchard, 1981; Vaughan & Silverstein, 1968).

**Figure 5.**
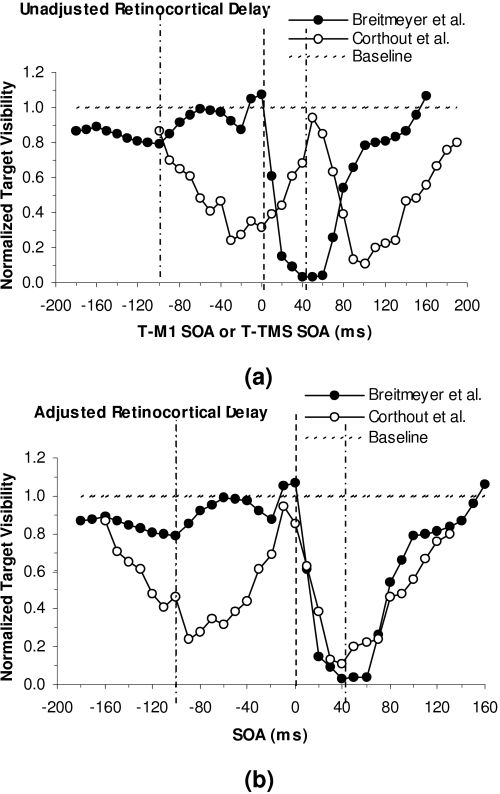
(a) Comparison of a typical masking function obtained in our laboratory using a visual para- or metacontrast mask and a typical masking function obtained by Corthout, Uttl, Ziemann et al. (1999) using a TMS pulse as a mask. Negative and positive SOAs indicate that the masks were presented before and after the target, respectively. Results are not adjusted for retinocortical transmission delay. (b) Same as preceding but with results adjusted for a 60-ms delay of cortical M activity due to retinocortical transmission time (Baseler & Sutter, 1997). (From Breitmeyer, Ro, Öğmen, 2004)

Figure 5a, taken from a recent study reported by Breitmeyer, Ro, and Öğmen (2004), shows the results of Corthout Uttl, Ziemann et al. (1999) again in comparison with paracontrast and metacontrast masking results obtained in our lab with visual masks. Note that here the TMS and visual para- and metacontrast masking maxima do not coincide. To make a proper comparison of the two sets of findings, in Figure 5b we shifted the visual masking results, so that the visual masking SOA of 0 ms aligned with a TMS SOA of 60 ms – for the following reasons. Assuming that the cortical effects of a TMS pulse occur at very short latencies (e.g. 10 ms or less), we took the value of 60 ms, based on results obtained by Baseler and Sutter (1997), as an estimate of the time delay (produced by sensory transduction and retino-geniculo-cortical transmission) separating the onset of the cortical responses to a visual mask presented to the retinas from the onset of the cortical TMS effect. Despite the use of different observers and procedures, the two studies yield masking functions that agree to a surprising extent, especially regarding the SOAs at which masking maxima occur. This result would be expected if the early and late TMS-suppression maxima and the para- and metacontrast masking maxima both correspond to the suppression of the early and late responses of V1 neurons, respectively.

**Figure 6.**
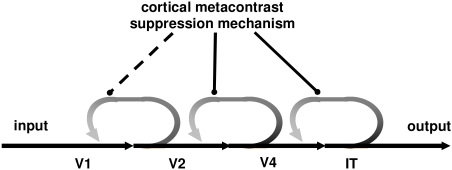
Schematic of hypothetical metacontrast suppression of reentrant activation in the cortical parvocellular (P) pathways.

This rather lengthy argument can now be summarized by the following schematic adopted from Rufin VanRullen’s work (VanRullen & Thorpe, 2002; VanRullen and Koch, 2003) and shown in Figure 6. A visual stimulus such as a target sets up an afferent feedforward sweep of activity that passes rapidly through several cortical levels of processing (e.g., V1 ➝ V2 ➝ V4 ➝ …). Each later level sends back re-entrant signals to the prior level(s) from which it received its feedforward drive, setting up a cascading reverberating loop of cortical activity. While paracontrast directly suppresses activity in the feedforward pathways (and thus, as argued above, indirectly also in the re-entrant sweep), metacontrast suppresses activity only in the re-entrant pathways. This is an important result since several theoretical approaches (Edelman, 1987, Edelman & Tononi, 2000, Zeki, 1993) and empirical findings (Pascual-Leone & Walsh, 2001) indicate that without the re-entrant signals, feature-specific contents of visual stimuli fail to register in consciousness.

### Neural-network modeling

For these reasons I maintain that neural-network models of backward *pattern* masking need to pay due attention to re-entrant cortical activations. Our updated REtinalCOrticalDynamics (RECOD) model (Breitmeyer & Öğmen, 2006; Öğmen & Breitmeyer, 2006), which Haluk Öğmen will cover more extensively, incorporates re-entrant feedback activity. Greg Francis’s (1997) BCS model also incorporates feedback from higher (cooperative) to lower (competitive) levels that potentially could assume the role of re-entrant signals. Of course, re-entrant activation is a prime component in the object-substitution (OS) model proposed by Vince Di Lollo, Jim Enns and co-workers (Di Lollo et al., 2000; Enns, 2004; Enns & Di Lollo, 1997).

Several recent findings, some from our own laboratories, however, do have implications for model building. One finding is the very existence of common-onset masking (Bischof, & Di Lollo, 1995; Di Lollo, Bischof, & Dixon, 1993; Di Lollo, Enns, & Rensink, 2000). Of course this finding is explained by the OS model. I think Bachmann’s PR model might also give an adequate account of the major aspects of common-onset-masking. While it has been suggested that some former models such as Bridgeman’s Hartline-Ratliff neural net may also give an account of common-onset masking (Bischoff & Di Lollo, 1995), Greg Francis’s recent work (Francis & Cho, 2006, submitted) indicates that models based on mask blocking may not. Without formal simulations, it is as yet not clear if and how the RECOD model could give an account.

In one of our studies (Öğmen, Breitmeyer, Todd, & Mardon, 2004), we have shown that there is a double dissociation between a stimulus’s effectiveness as a mask and its visibility. That is to say, we demonstrated that one can obtain masking of a target even though the visibility of the primary metacontrast mask is itself suppressed by a secondary one. This demonstrates Dissociation 1: the neural processes or mechanisms contributing to the masking effectiveness of the primary mask can be activated without at the same time activating the processes leading to the conscious registration of the primary mask. Conversely, we also showed that a highly visible primary mask nonetheless can be rendered ineffective in its suppression of a target’s visibility. This demonstrates Dissociation 2: the neural processes or mechanisms contributing to the visibility or conscious registration of the primary mask can be activated without activating the processes supporting its effectiveness as a mask. This shows that a transient stimulus activates two distinct neural processes: one responsible for its visibility; the other, for its effectiveness as a mask. We have shown further that the former and the latter processes have contrast gain functions that resemble those of the parvo- and magnocellular (P and M) pathways, respectively. Although I need not be wedded to a dual-channel model, we take this as undeniably strong evidence that the dual-channel, sustained transient model of masking is still much alive and vigorous, at least within an updated P and M framework. For that reason I remain theoretically true to this model. To paraphrase one of my favorite writers, Umberto Eco, monogamy to the dual-channel model does not mean lack of libido.

**Figure 7.**
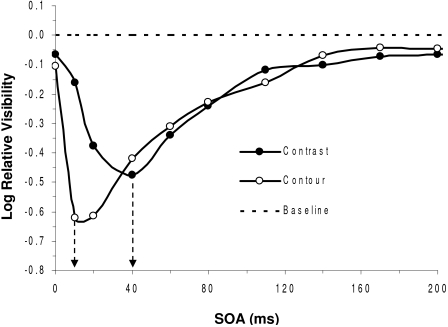
Metacontrast contour and surface-contrast suppression as a function of stimulus onset asynchrony (SOA). (Adapted after Breitmeyer et al., 2006)

In another study (Breitmeyer, Kafaligönül, Öğmen, Mardon, Todd, & Ziegler, 2006), we also have shown that metacontrast masking can separately affect contour and surface properties of visual objects. In this study, observers were required to judge the target either with regard to its contour detail or else its surface brightness. The results, shown in Figure 7, show that two distinct metacontrast functions are obtained for these two correspondingly distinct tasks. Both tasks yielded typical U-shaped metacontrast functions. However, while the contour task yielded optimal masking at a short SOA of 10 ms, the brightness task yielded optimal masking at a higher SOA of 40 ms. This indicates that an object’s surface brightness is processed about 30 ms later than its contour. These findings are consistent with several theoretical and empirical results. For one, Grossberg and colleagues (Cohen & Grossberg, 1984; Grossberg, 1994; Grossberg & Yazdankbakhsh, 2005) in their FAÇADE and more recent LAMINART model have posited two separate processes, the Boundary Contour System (BCS), which processes contour edges or boundaries, and the Feature Contour System (FCS), which processes the surface features filling in the area between contour boundaries. In Grossberg’s (1994) theory the BCS and FCS have their neural correlates in the separate form-processing P-interblob and surface-processing P-blob cortical pathways (De Yoe & van Essen, 1988; Xiao, Wang, & Felleman, 2003). Moreover, Lamme, Rodriguez-Rodriguez, & Spekreijse (1999) recently have shown that the surface-defining response in V1 lags the contour-defining response by about 40 ms, a value consistent with the 30 ms lag estimated from our metacontrast findings.

It is not clear whether Francis’s BCS model can account for these results, since it is premised on only the BCS component of Grossberg’s (1994; Grossberg & Yazdankbakhsh, 2005) FAÇADE or LAMINART model. Foreseeably the BCS model will have to be complemented with an FCS component in order to account for the separate suppression of contour and surface features. The RECOD model has already been adapted to account for these findings simply by assuming that a target’s contour and surface information are separately processed by the P-interblob and the slower P-blob cortical pathways, respectively. Bachmann’s PR model could also account for these results, by adopting the same assumptions that we have adopted. In a modified PR model, this assumption could be instantiated via two separate specific afferent processes, one corresponding to the contour-forming process, the other to the slower surface-defining process. I am not sure what, if any, problem these results might pose for the OS model. It depends on what constitutes or is meant by an *object*. Is it represented as a unitary, holistic Gestalt-like entity or can one envisage it as an ensemble of conjoined yet distinct features or perhaps both? Indeed, recent evidence reported by Gelattly, Pilling, Cole, & Skarratt (2006) suggests that OS masking may occur at a feature as well as an object level of representation. Since OS masking is assumed to be intimately tied to attention (Enns & Di Lollo, 1997; Di Lollo et al., 2000), this feature-specific OS masking is entirely consistent with other recent reports of feature-based (as compared to object-based) attention (Hayden & Gallant, 2005; Nobre, Rao & Chelazzi, 2006) In view of these findings, I think that a clear theoretical statement specifying the relation between features and objects may need to be spelled out in the OS model.

## What Next?

As with weather forecasting, forecasting developments in any field of research is an inexact exercise. The safest bet is that things will be much the same tomorrow as today. Easier is the task of posing questions that might define some of the paths that future developments take. I think two key questions are: What *unique* aspects distinguish one model from another? And what aspects of one model can map onto homologous or analogous aspects of another? For instance, I see the activation of the retino-reticular-thalamic system in the PR model as a unique aspect not shared by other models; and so far the activation of reentrant processes has been unique to the OS model. On the other hand, a form of object substitution per se (beyond mere phenomenological description) seems to be common to the PR and the OS model. Greg Francis (Francis & Cho, 2006, submitted; Francis & Herzog, 2004) is currently examining some of the abstract, formal properties common or unique to several models. This sort o theoretical work can be very useful in answering these two questions. A third question is: In view of ever new empirical findings, how might the various models be updated? What aspects should be retained? What ones can be discarded? What new components must be added? In the prior section I have already listed some empirical findings that indicate a need for updating models. A fourth question is: Is it possible that such updates might formally converge on some sort of *supermodel*? Answers to the prior questions may suggest such a convergence that is more than the logical intersection, yet less than the eclectic union, of the extant models. On the other hand a supermodel might be radically different from any of the current ones.

**Figure 8.**
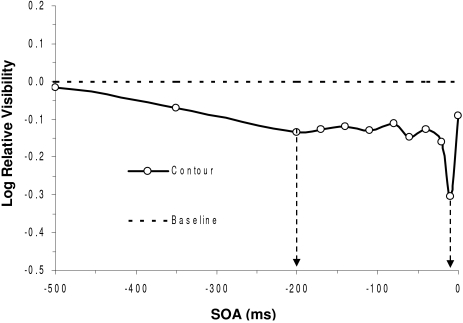
Paracontrast contour suppression as a function of SOA. Note the two minima in target contour visibility at -200 and -10 ms. (Adapted after Breitmeyer et al., in press)

Another, more empirically fruitful question concerns the neural correlates of masking and specifically the neural mechanisms that contribute to masking. I have already touched on some aspects of the question in prior sections. In terms of paracontrast, it seems clear to me that Macknik and Livingstone’s (1998) contributions are very telling. Paracontrast results from suppression of the early V1 response component, and presumably of the cortical feedforward drive. Exactly how such suppression is instantiated remains to be worked out. Some of it could be due to simple center-surround antagonism of classical receptive fields not only at cortical levels but also at subcortical levels, as originally proposed by Breitmeyer and Ganz (1976). Since the surround response lags the center response by 10-30 ms, one would expect optimal paracontrast at a very short negative SOA. Figure 8 shows a typical result from a recent studies (Breitmeyer et al., 2006) conducted in our laboratories. Here a contour discrimination task was used to index masking. Note that indeed a local maximum in the masking effect occurs at an SOA of -10 ms. This would be consistent with center-surround interactions within antagonistically organized receptive fields. However, note also that there is a second maximal masking effect at an SOA of roughly 200 ms, more in line with neurophysiological findings reported by Macknik and Livingstone (1998) and with prior psychophysical findings (Cavonius & Reeves, 1983; Scharf & Lefton, 1970). This effect cannot be explained by the center-surround antagonism of classically defined receptive fields. Some other sort of process, perhaps akin to the longer lasting cortical inhibition reported by several investigators (Berman, Douglas, Martin, & Whitteridge, 1991; Connors, Malenka, & Silva, 1988; Nelson, 1991) is involved. At any rate, I think more work might elucidate the various mechanisms of paracontrast.

With regard to metacontrast, Haynes, Driver et al’s (2005) fMRI results are suggestive. Metacontrast yields a decorrelation between the earlier activity in V1 and the later activity in the fusiform gyrus. The questions remaining to be answered are: What is the mechanism or process by which such decorrelation is produced? And where in the V1-to-fusiform gyrus pathway does this process exert its effects. I am not sure what sorts of neuroscientific methods could answer these questions, but they certainly deserve attempts at an answer. Partial answers already exist. I believe the work of Steve Macknik and Susana Martinez-Conde and colleagues (Macknik & Martinez-Conde, 2004; Tse, Martinez-Conde, Schlegel, & Macknik, 2005) indicate that the suppressive mechanisms occur at cortical binocular levels of processing primarily beyond areas V1/V2. At any rate, I see a lot of work still needing to be done before we better understand the neural processes underlying metacontrast.

**Figure 9.**
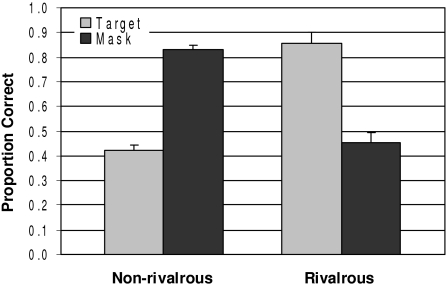
Target and mask visibilities (in proportion correct stimulus identification) under nonrivalrous (standard dichoptic) viewing of the target and the mask and under viewing in which the visibility of the mask is suppressed during binocular rivalry.

Finally, it is important to note that masking has become one of the several methods for exploring NCCs and NCUs. The other ways include binocular-rivalry suppression, the attentional blink (AB), change blindness, inattentional blindness, motion induced blindness, generalized flash suppression, and crowding or lateral masking. While these are all useful ways of “skinning” consciousness, they do not yield equivalent results. Figure 9 shows results we (Breitmeyer, Öğmen, & Koç, 2005) recently obtained in which metacontrast masking was studied under nonrivalrous dichoptic viewing in comparison to when the eye to which the mask was presented was in the suppressed phase of binocular rivalry. Note that in the nonrivalrous condition, the results indicate low visibility of the target and high visibility of the mask, a result typical under standard dichoptic viewing of the stimuli (Kolers & Rosner, 1960; Schiller & Smith, 1968, Weisstein, 1971). However, in the rivalrous condition, the target’s visibility is no longer suppressed, while that of the mask is. This target recovery or disinhibition in the rivalrous condition indicates that not only the neural processes responsible for the visibility of the mask but also those responsible for its effectiveness as a suppressor of the target are suppressed during binocular rivalry. In other words, here we do not obtain the aforementioned dissociation between the two distinct mask-activated neural processes. This indicates that binocular-rivalry *can* suppress the metacontrast mechanism and thus that binocular-rivalry suppression and metacontrast suppression work at different *functional* levels of processing. In some sense binocular-rivalry suppression is functionally prior to metacontrast suppression. How this might translate into underlying neurophysiology is hard to assess. However, at first glance the priority of binocular-rivalry relative to metacontrast suppression appears consistent with a) the results reported by Macknik and Martinez-Conde (2004), Haynes Deichmann, and Rees (2005), and Tse et al. (2005) showing that metacontrast and visual pattern masking occur at fairly late levels in the cortical visual pathway and 2) the recent findings showing neural signatures of binocular rivalry suppression in humans as early as the lateral geniculate nucleus (Haynes, Deichmann et al., 2005, Wunderlich, Schneider, & Kastner, 2005). For these reasons, I believe that by looking at how masking relates to other psychophysical “blinding” methods and how any emerging differences correlate with differences in neuro- and electrophysiological findings or in brain imaging results one can more clearly delimit the elusive NCCs and NCUs in vision.
